# Valorization of Alkaline Peroxide Mechanical Pulp by Metal Chloride-Assisted Hydrotropic Pretreatment for Enzymatic Saccharification and Cellulose Nanofibrillation

**DOI:** 10.3390/polym11020331

**Published:** 2019-02-14

**Authors:** Huiyang Bian, Xinxing Wu, Jing Luo, Yongzhen Qiao, Guigan Fang, Hongqi Dai

**Affiliations:** 1 Jiangsu Co-Innovation Center of Efficient Processing and Utilization of Forest Resources, Nanjing Forestry University, Nanjing 210037, China; hybian1992@njfu.edu.cn (H.B.); xinxingwu@njfu.edu.cn (X.W.); luojing@njfu.edu.cn (J.L.); qiaoyz0210@163.com (Y.Q.); 2 College of Chemical Engineering, Nanjing Forestry University, Nanjing Forestry University, Nanjing 210037, China; 3 China Institute of Chemical Industry of Forestry Products, Chinese Academy of Forestry, Nanjing 210042, China; fangguigan@icifp.cn

**Keywords:** hydrotropic treatment, metal chloride, delignification, enzymatic saccharification, lignocellulosic nanofibrils

## Abstract

Developing economical and sustainable fractionation technology of lignocellulose cell walls is the key to reaping the full benefits of lignocellulosic biomass. This study evaluated the potential of metal chloride-assisted *p*-toluenesulfonic acid (*p*-TsOH) hydrolysis at low temperatures and under acid concentration for the co-production of sugars and lignocellulosic nanofibrils (LCNF). The results indicated that three metal chlorides obviously facilitated lignin solubilization, thereby enhancing the enzymatic hydrolysis efficiency and subsequent cellulose nanofibrillation. The CuCl_2_-assisted hydrotropic pretreatment was most suitable for delignification, resulting in a relatively higher enzymatic hydrolysis efficiency of 53.2%. It was observed that the higher residual lignin absorbed on the fiber surface, which exerted inhibitory effects on the enzymatic hydrolysis, while the lower lignin content substrates resulted in less entangled LCNF with thinner diameters. The metal chloride-assisted rapid and low-temperature fractionation process has a significant potential in achieving the energy-efficient and cost-effective valorization of lignocellulosic biomass.

## 1. Introduction

Lignocellulose biomass is an abundant and sustainable, carbon-neutral resource. It can be converted into different high-value-added products (e.g., biofuels and nanocellulose). Biofuels, as a promising renewable energy alternative to fossil fuels, are usually derived from relatively inexpensive, abundant, and renewable agricultural or industrial byproducts, such as wheat straw, corn stover, or forestry residues [1,2,3]. Nanocellulose, as a green alternative to artificial polymers, exhibits excellent properties, such as high elastic modulus, high specific surface area, optical transparency, low thermal expansion coefficient, and chemical reactivity [4,5,6]. However, lignocellulose consists of carbohydrate polymers (cellulose and hemicellulose) and an aromatic polymer (lignin), which are tightly bonded together by ester and ether linkages [7]. The complexity of these components and their arrangement makes the cell wall naturally resistant, therefore making pretreatment necessary for its deconstruction or fractionation, which is the key to achieving a green, biobased economy.

To date, various pretreatment approaches have been investigated on economical and sustainable deconstruction or fractionation of lignocellulose cell walls [8,9,10,11,12]. These strategies facilitate the exposure of more cellulose and hemicellulose for enzymatic saccharification or cellulose nanofibrillation. Delignification is critical for successful cell wall deconstruction because lignin is a major cell wall polymer, with the middle lamella lignin acting as a glue to hold cells together in plant biomass [13]. Conventional delignification methods include alkaline wood pulping (120 °C or higher for 2 h), kraft pulping (120–160 °C for 1–2 h), and aromatic salts pretreatment (170 °C for 2 h) [14,15,16,17]. However, the high demand of reaction temperature and pressure and long reaction periods constitute major challenges in the delignification process, which increase the cost of downstream production. It has been demonstrated that the hydrotropic *p*-toluenesulfonic acid (*p*-TsOH) can dissolve approximately 90% of poplar wood (NE222) lignin at 80 °C in 20 min [18]. Unlike the traditional approach of alkaline and acid sulfite cooking by the extraction of lignin at the expense of degrading a considerable amount of another components, this hydrotropic pretreatment can maintain cellulose and hemicellulose for a low energy input and sustainable production of valuable building blocks, such as sugars, fibers, and wood-based nanomaterials [19,20,21,22]. Limited attention, however, has been paid to discuss the lignin redistribution on the fiber surface after *p*-TsOH pretreatment and its effect on subsequent enzymatic saccharification or lignocellulose nanomaterials production.

Note that some metal chlorides can effectively hydrolyze carbohydrates into useful chemicals and exhibit higher catalytic activity than inorganic acids [23]. Some studies have reported on the production of ethanol or cellulose nanocrystals using metal chlorides to pretreat rice straw or enhance cellulose hydrolysis [24,25,26,27,28]. In order to reduce the acid and energy consumption, three inorganic chlorides (FeCl_3_, AlCl_3_, and CuCl_2_) were introduced into hydrotropic pretreatment of alkaline peroxide mechanical pulp (APMP) fiber at a relatively low acid concentration and reaction temperature in this work. The enzymatic digestibility of cellulose after pretreatment was investigated. The enzyme-treated residual solids were further fibrillated to produce lignocellulosic nanofibrils (LCNF). The amount of lignin retained in hydrolyzed lignocellulose can be tuned by selecting various metal chlorides as the catalysts, thereby endowing LCNF with different morphologies and physicochemical properties. Our research includes delignification, enzymatic efficiency, and LCNF physicochemical properties to gain insight into the valorization of APMP. Compared to pure *p*-TsOH hydrolysis, the metal chloride-assisted hydrotropic pretreatment can achieve equivalent delignification using lower acid concentration and temperature, providing useful information in economic and environmental utilization of lignocellulosic biomass for biofuels and materials.

## 2. Materials and Methods 

### 2.1. Materials

Alkaline peroxide mechanical pulp (APMP) fiber was provided from Huatai Paper Co., Ltd., Shandong, China. *p*-Toluenesulfonic acid (*p*-TsOH) was the analytical reagent and purchased from LiFeng Chemical Reagent Co. Ltd., Shanghai. Ferric chloride hexahydrate (FeCl_3_·6H_2_O), aluminum chloride hexahydrate (AlCl_3_·6H_2_O), and copper chloride dehydrate (CuCl_2_·2H_2_O) were purchased from Aladdin Co. Ltd., Shanghai, China. Cellulase (Cellic^®^ CTec2) was kindly provided by Novozymes North America (Franklinton, NC, USA), with filter paper activity of 250.0 FPU/mL and cellubiase activity of 2731 U/mL.

### 2.2. Metal Chloride-Assisted Hydrotropic Pretreatment

Briefly, 5 g of APMP fibers were hydrolyzed in 70% (w/w) concentration *p*-TsOH solution with 0.1 mM three different metal chlorides (FeCl_3_·6H_2_O, AlCl_3_·6H_2_O and CuCl_2_·2H_2_O) at 65 °C with a liquid to solid mass ratio of 10:1 (g/g). The fiber suspension was constantly agitated using a mixer at 500 rpm for 35 min. The reaction was immediately quenched by adding 100 mL of DI water, then the hydrolysate was separated by vacuum filtration through a filter paper and the residual solids were dialyzed until the pH of the water no longer changed. For comparison, hydrotropic pretreatment without adding metal chloride was also performed under the same condition.

### 2.3. Enzymatic Saccharification

Enzymatic hydrolysis of pretreated samples was carried out in 0.05 M sodium citrate buffer (pH 4.8) with a substrate concentration of 1% (w/v) on a rotary shaker (50 °C, 150 rpm). The enzyme (Cellulase, Cellic^®^ CTec2) loading was 20 FPU/g glucan during the hydrolysis experiment. Samples were taken at different time points (2, 4, 6, 12, 24, 48 and 72 h), incubated in boiling water to inactivate the enzymes, then centrifuged to remove residual solids. To quantify monosaccharides in hydrolysate, the supernatants were diluted for HPLC (high performance liquid chromatography) analysis. All experiments were carried out in duplicate and results were presented as the average value of two replicated tests.

### 2.4. Lignocellulosic Nanofibrils Production

**Figure 1:**
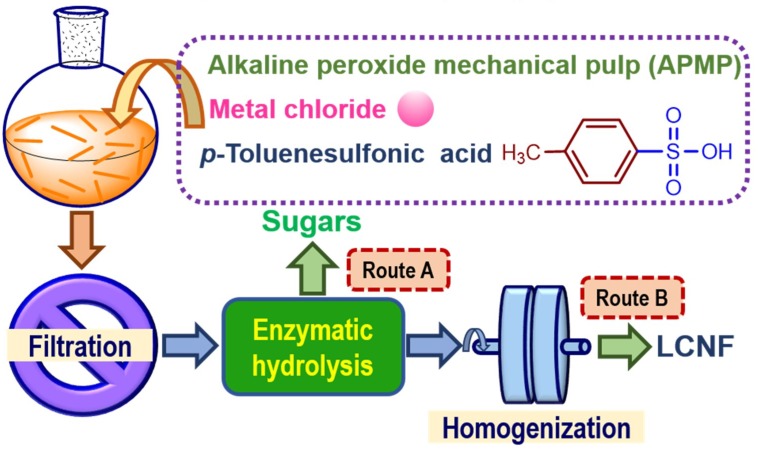
Routes for the valorization of alkaline peroxide mechanical pulp (APMP) using metal chloride-assisted hydrotropic pretreatment. Route A, enzymatic saccharification; Route B, lignocellulosic nanofibrils production.

All enzyme treated residual samples were mechanically fibrillated for producing LCNF using a high pressure homogenizer (FB-110Q, LiTu Mechanical Equipment Co., Ltd, Shanghai, China) with an operation pressure of 600 bar for 5 passes. A schematic flow diagram describing the routes for the valorization of APMP using metal chloride-assisted hydrotropic pretreatment is shown in Figure 1.

### 2.5. Characterization

The chemical compositions (including structural polysaccharides and lignin) of all samples were measured using the National Renewable Energy Laboratory (NREL) standard method [29]. Monosaccharides and inhibitors in this study were analyzed by the HPLC system (Agilent 1260 series, Agilent Technologies, Santa Clara, CA, USA). Recovery yields of carbohydrates (glucan and xylan), degree of delignification, and enzymatic efficiency were calculated according to the following equations:

(1) $$\mathrm{Glucan}\;\mathrm{recovery}\;\mathrm{yield}\;\left(\%\right)=\frac{\mathrm{glucan}\;\mathrm{in}\;\mathrm{pretreated}\;\mathrm{APMP}\;\left(g\right)}{\mathrm{glucan}\;\mathrm{in}\;\mathrm{raw}\;\mathrm{APMP}\;\left(g\right)}\times 100\%$$

(2) $$\mathrm{Xylan}\;\mathrm{recovery}\;\mathrm{yield}\;\left(\%\right)=\frac{\mathrm{xylan}\;\mathrm{in}\;\mathrm{pretreated}\;\mathrm{APMP}\;\left(g\right)}{\mathrm{xylan}\;\mathrm{in}\;\mathrm{raw}\;\mathrm{APMP}\;\left(g\right)}\times 100\%$$

(3) $$\mathrm{Delignification}\;\left(\%\right)=1-\frac{\mathrm{lignin}\;\mathrm{in}\;\mathrm{pretreated}\;\mathrm{APMP}\;\left(g\right)}{\mathrm{lingin}\;\mathrm{in}\;\mathrm{raw}\;\mathrm{APMP}\;\left(g\right)}\times 100\%$$

(4) $$\mathrm{Enzymatic}\;\mathrm{efficiency}\;\left(\%\right)=\frac{\mathrm{glucose}\;\mathrm{in}\;\mathrm{enzymatic}\;\mathrm{hydrolyzate}\;\left(g\right)}{\mathrm{inital}\;\mathrm{glucose}\;\mathrm{in}\;\mathrm{substrate}\;\left(g\right)}\times 100\%$$

The morphologies of the acid hydrolyzed samples were observed using scanning electron microscopy (SEM) and laser scanning confocal fluorescence microscopy (LSCM). For SEM analyses, samples were sputter-coated with gold to provide adequate conductivity. Images were observed and recorded using a SEM system (Quanta 200, FEI, Hillsboro, OR, USA). For LSCM analyses, samples were diluted in DI water and deposited onto clean glass substrates. A Zeiss LSM 710 laser scanning confocal microscopy was used with excitation lased at Ar 488 nm over an emission range of 490 to 560 nm and 40× C-Apochromat objective (1.1 W NA) zoom to acquire multichannel fluorescence image.

LCNF morphology was observed using atomic force microscopy (AFM, Dimension Edge, Bruker, Germany). Samples were diluted to a solids consistency of 0.01 wt %, deposited onto clean mica substrates and air-dried overnight at room temperature. AFM topographical images were obtained in tapping mode at 300 kHz using a standard silicon cantilever and a tip with a radius of curvature of 8 nm. Fibril height distribution was measured using Gwyddion software (Department of Nanometrology, Czech Metrology Institute, Jihlava, Crezh Republic, 64-bit). The surface charges of LCNF samples were measured using a zeta potential analyzer (Malvern Instruments Ltd., Zetasizer NanoZS, Malvern, UK). Five measurements were determined and the results were presented as average values. The XRD patterns were measured using an Ultima IV diffractometer at a voltage of 40 kV and a current of 30 mA (Rigaku Corp., Tokyo, Japan). Scattering radiation was detected in a 2θ range from 10° to 40° in steps of 0.02°. The crystallinity index (CrI) of LCNF samples was calculated in accordance with the Segal method (without baseline substrate) as described previously [30]. The FTIR spectra of LCNF samples were obtained by a Fourier-transform infrared spectrometer (Nicolet 380, Waltham, MA, USA). Samples were ground into powders and blended with KBr powder, then pressed into a disk at 30 MPa. The spectrum for each sample was recorded in the region of 4000–500 cm^−1^. The thermal properties of the LCNF samples were analyzed using a thermogravimetric analyzer (Q5000IR, TA instruments, New Castle, DE, USA). Samples of approximately 5 mg were heated from 50 to 600 °C under a 20 mL/min high-purity nitrogen stream using a heating rate of 10 °C/min.

## 3. Results

### 3.1. Component Change of the Fibers after Pretreatments

**Figure 2:**
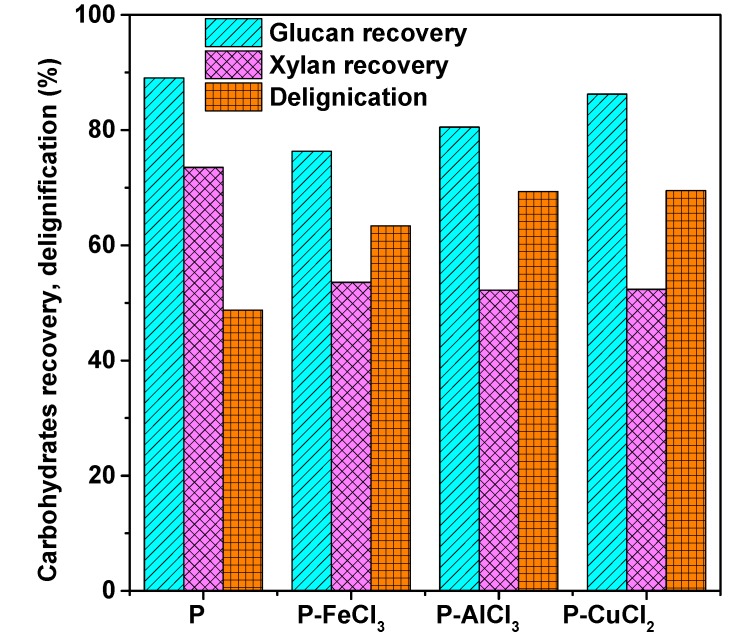
Carbohydrate (glucan and xylan) recovery and lignin removal rate of pretreated materials after different metal chloride-assisted hydrotropic pretreatments.

Component change of lignocellulosic biomass is helpful to assess the influence of pretreatment. The chemical compositions and yields of the APMP from fractionation runs under different metal chloride-assisted hydrotropic pretreatments are summarized in Table 1, along with the glucose and xylose concentrations in the spent liquors. The results indicated that the solid yields obviously decreased from 80.18% to 65.94%, 63.32%, and 64.47%, respectively, after the addition of metal chlorides (FeCl_3_·6H_2_O, AlCl_3_·6H_2_O, and CuCl_2_·2H_2_O) during hydrotropic pretreatment. Metal chlorides could have formed hydrated complexes in aqueous solution and coordinated with the glycosidic oxygen of cellulose. These metal cations acting as Lewis acids could have helped to break down the glycosidic linkages and then facilitate the cellulose hydrolysis process, resulting in the mass loss of biomass [31]. As shown in Figure 2, the low recovery yield of xylan and high lignin removal rate (expressed as delignification) also confirmed the low solid yield of pretreated APMP. However, glucan losses were only at approximately 10%, suggested that the *p*-TsOH had the great selectivity of solubilizing lignin. It is worth noting that FeCl_3_-assisted acid hydrolysis showed strong catalytic activity to facilitate the hydrolysis process, however it also had the lowest degree of delignification compared with the other metal chlorides. This result may be due to the competition between ionic bonds and intermolecular forces during the delignification. To be specific, it has been known that the *p*-TsOH can readily provide protons in an aqueous solution to break glycosidic, ether, and ester bonds in carbohydrates and lignin–carbohydrate complexes [18]. The lipophilic nonpolar part (toluene moiety) of the *p*-TsOH molecule can shield the separated lignin by π–π stacking or hydrophobic interaction to form micellar aggregates to prevent the aggregation of lignin, while the hydrophilic end (sulfonic acid moiety) is directed outward to the water for effective dissolution [32]. However, metal cations possess higher catalytic activity and ability to attract electron pairs, which can capture the hydrophilic end (sulfonic acid moiety) to form strong ionic bonds. The separated lignin undergoes intermolecular condensation, or recondensation in the absence of a hydrophobic solute, to be redeposited onto a solid substrate [18].

### 3.2. Effect of Treatments on Fiber Morphology and Microstructure

**Figure 3:**
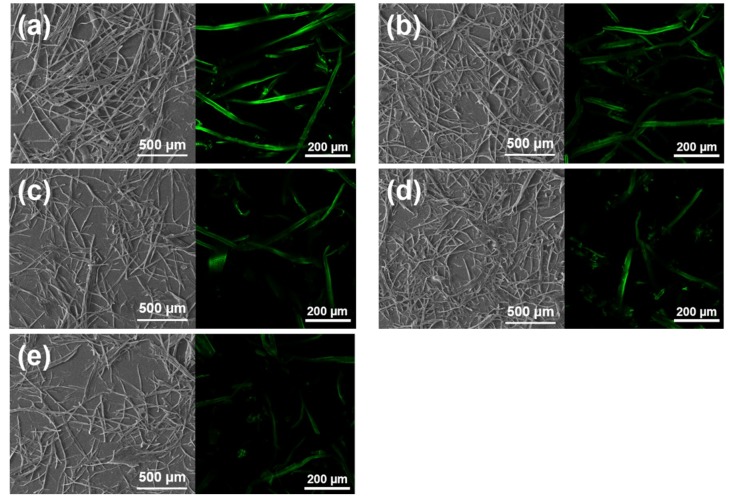
Scanning electron image and confocal microscopy image of alkaline peroxide mechanical pulp with different metal chloride-assisted hydrotropic pretreatments. (**a**) APMP; (**b**) APMP–P; (**c**) APMP–P–FeCl_3_; (**d**) APMP–P–AlCl_3_; (**e**) APMP–P–CuCl_2_.

The morphological changes of APMP during the different metal chloride-assisted hydrotropic pretreatments were observed by scanning electron and confocal microscopy techniques (Figure 3). The left row of each figure shows the SEM images of original and hydrolyzed APMP fibers. After the *p*-TsOH treatment, the obtained solid residues were substantially shortened compared to the original APMP fibers. The fiber shortening was more pronounced for metal chloride-assisted hydrotropic pretreatment than pristine *p*-TsOH, due to the presence of metal cations that facilitated carbohydrate degradation. Since no severe pretreatment (temperature = 65 °C) was applied, it was likely that partial lignin distributed itself on the fiber surface. To track the residual lignin distribution, confocal imaging was performed on feedstock and hydrolyzed samples. The green autofluorescence in the right row of each figure is from lignin [33]. The APMP fiber with highest lignin content had homogenous lignin distribution on the fiber surface. After hydrotropic pretreatments with or without metal chloride, partial lignin was removed from the fiber surface due to the presence of *p*-TsOH. Among three different metal cations, the APMP–P–CuCl_2_ fiber exhibited the least green autofluorescence, i.e., the minimal residual lignin content, which was consistent with the data from chemical composition analysis (Table 1). The lignin content and redistribution may affect the downstream production (enzymatic hydrolysis and LCNF production), as discussed in the following text.

### 3.3. Enzymatic Digestibility of Acid-Hydrolyzed Fraction

**Figure 4:**
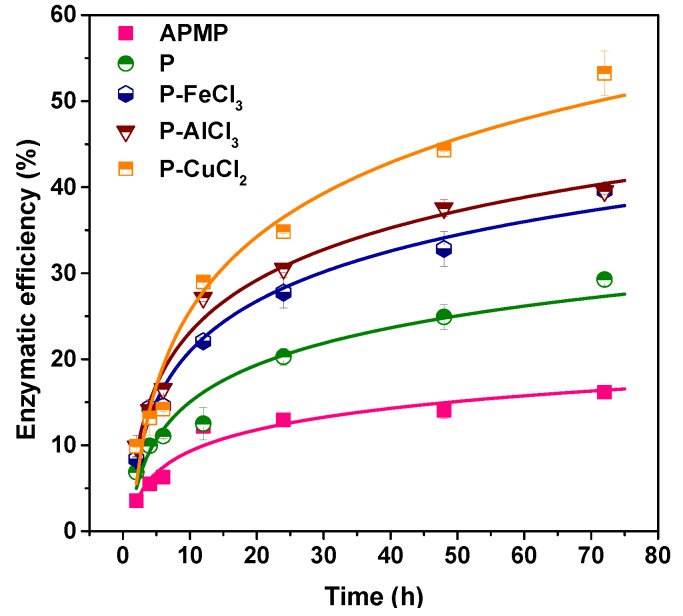
Time-dependent enzymatic efficiency of fractionated APMP fibers from various metal chloride-assisted (Fe^3+^, Al^3+^, and Cu^2+^) hydrotropic pretreatment under constant cellulase (Cellic^®^ CTec2) loading of 20 FPU/g glucan.

To evaluate the effects of different metal cations on APMP sugar generation, all pretreated residues were used as the substrates for enzymatic hydrolysis. The enzymatic efficiency was only 16.1% for the raw material. After various metal chloride-assisted (Fe^3+^, Al^3+^, and Cu^2+^) hydrotropic pretreatments, the enzymatic efficiency was found to be greater than that of the APMP without pretreatment (Figure 4). For example, the 72-h enzymatic hydrolysis efficiency of P–APMP and P–CuCl_2_–APMP were 29.3% and 53.2%, respectively, which were 1.8 and 3.3-fold higher than that of the raw material without pretreatment. It has been reported that the “blocking effect” of xylan was one of the major obstacles which limited the enhancement of enzymatic efficiency [34]. The xylan recovery of P–APMP was higher than those with metal chloride-assisted hydrotropic pretreatments, therefore the enzymatic efficiency was relatively low. However, it should be noted that although the xylan recovery of the three metal chloride-assisted treated APMP fibers were almost the same (approximately 53%), their enzymatic efficiencies were significantly different. This phenomenon might be due to the presence of residual lignin, which had a negative effect on the yields of enzymatic hydrolysis. It has been reported that iron–lignin complexation exerted inhibitory effects on the enzymatic hydrolysis of autohydrolyzed biomass [35]. Therefore, although different *p*-TsOH pretreatments removed partial lignin, the residual lignin absorbed on the fiber surface still led to the formation of a lignin–enzyme complex, which was considered to be ineffective for the enzymatic hydrolysis process. In view of all enzymatic hydrolysis data, a conclusion can be made that the removal of plenty of hemicelluloses and lignin during the pretreatment process was highly related to the substrate enzymatic efficiency.

### 3.4. Properties of LCNF

**Figure 5:**
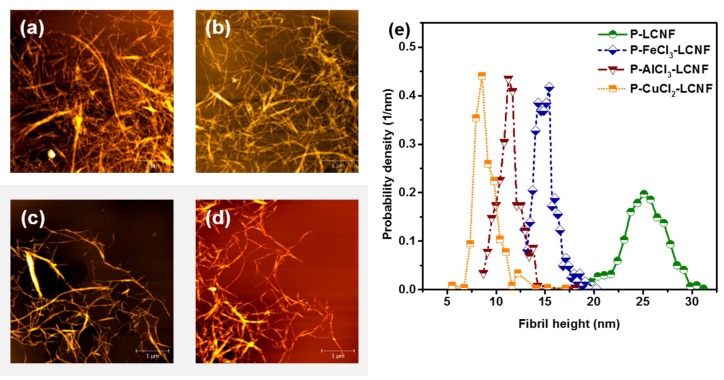
Effect of different metal chloride-assisted hydrotropic pretreatment on the morphologies of the resulting lignocellulosic nanofibrils (LCNF) measured by atomic force microscopy (AFM). All scale bar = 1 µm. (**a**) P–LCNF, mean height = 25.2 nm; (**b**) P–FeCl_3_–LCNF, mean height = 15.1 nm; (**c**) P–AlCl_3_–LCNF, mean height = 11.5 nm; (**d**) P–CuCl_2_–LCNF, mean height = 9.0 nm; (**e**) AFM measured LCNF height probability density distributions.

Subsequent mechanical fibrillation of various partially enzyme-hydrolyzed samples using high pressure homogenizer produced LCNF with varied morphologies, as shown by AFM in Figure 5. Some small, globular-shaped lignin particles were visible, especially at P–LCNF without metal chloride-assisted fractionation [36]. Introducing metal ions during hydrotropic pretreatments resulted in less entangled LCNF with thinner diameters as observed from the AFM images and AFM measured height distributions (Figure 5e). Furthermore, the distribution of P–metal chloride–LCNF became narrower or more uniform as different metal chlorides were added. The number averaged fibril heights were 25.2, 15.1, 11.5, and 9.0 nm, respectively, for P–LCNF and the three different P–metal chloride (FeCl_3_, AlCl_3_, and CuCl_2_)–LCNFs (Table 2). This was due to the fact that higher residual lignin content in P–APMP was considered to be the barrier to impede mechanical fibrillation, consistent with the results found in the literature using medium-density fiberboard and agricultural waste to produce LCNF [20,21].

**Figure 6:**
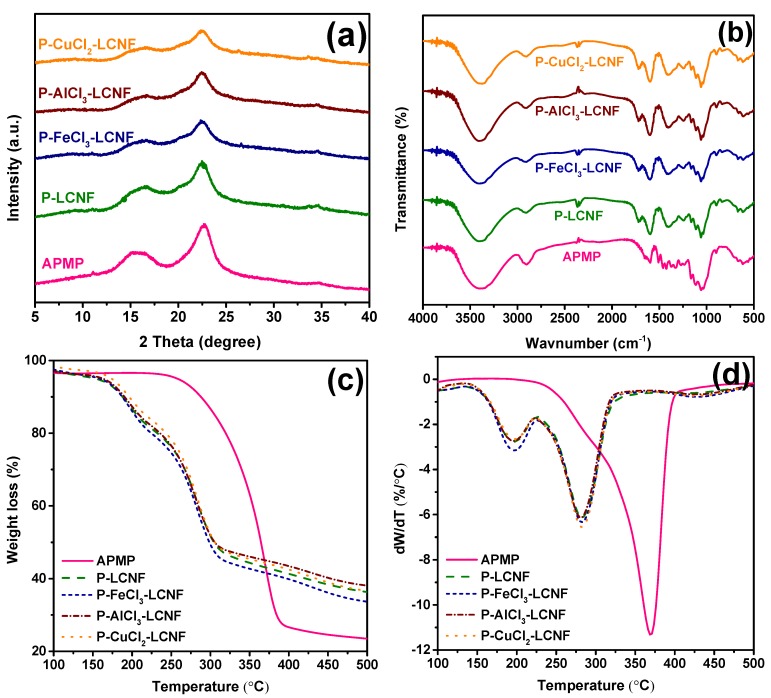
Comparisons of raw material and lignocellulosic nanofibrils produced from different metal chloride-assisted hydrotropic pretreatment. (**a**) XRD diffractogram; (**b**) FTIR spectra; (**c**) TGA weight loss; (**d**) TGA temperature derivative weight loss.

The XRD patterns of both raw and resulting LCNFs are presented in Figure 6a. XRD analysis was conducted to investigate the crystalline features of the fibers and the relationship between structures and properties. Two main characteristic peaks at about 2θ = 16.4° and 22.6° corresponded to the (100) and (200) reflection planes, indicating that only cellulose I structure was present in all samples [37]. These results suggest various treatments did not destroy or alter the inherent crystal structure of cellulose. The crystallinity index (CrI) of all LCNF samples was higher than the APMP (Table 2), despite the fact that enzymatic hydrolysis and mechanical fibrillation could break up cellulose crystals. This is because hydrotropic pretreatment dissolved substantial amounts of amorphous lignin and hemicellulose (mainly xylan), as also revealed by the compositional analysis of the substrates (Table 1). 

The zeta-potentials of all LCNF samples were not much different (Table 2), suggesting that metal ions (Fe^3+^, Al^3+^, and Cu^2+^) only acted as catalysts to help to break down the glycosidic linkages and did not induce the formation of new charged functional groups in undissolved carbohydrates. This can be verified from the comparison of the FTIR spectra between the APMP and LCNF samples (Figure 6b). Only small variations in the lignin region were due to the removal of partial lignin during the hydrotropic pretreatment.

The thermal behaviors of raw APMP fiber and resulting LCNF samples are presented in Figure 6c,d. The maximum degradation temperature (T_max_) was determined from the derivative thermogravimetric (DTG) peak at which the maximum decomposition rate was obtained. Lignin presents a wide range of decomposition temperatures, is known to be thermally more stable than cellulose and hemicellulose, and the increased surface area from enzymatic hydrolysis and nanofibrillation may have facilitated increased thermal degradation. Therefore, the APMP fiber with a lignin content of 20.65% showed excellent thermal stability with a T_max_ of 368.7 °C, higher than those LCNF samples with a lower lignin content. It should be noted that the degradation of LCNF showed two peaks evident from DTG curves (Figure 6d). The first peak can probably be attributed to the removal of enzyme that remained during the enzymatic hydrolysis. The second one in the dW/dT plot was associated with the degradation of residual lignin [38]. Although P–CuCl_2_–LCNF had the lowest lignin content among three different P–metal chloride–LCNFs, it exhibited similar thermal stability. The reason, perhaps, is due to the higher crystalline structure (CrI value in Table 2) for the P–CuCl_2_–LCNF sample, which required higher degradation temperature.

## 4. Conclusions

A rapid and efficient approach was performed to remove lignin at a low temperature, in which three metal chlorides (FeCl_3_, AlCl_3_, and CuCl_2_) were introduced into the hydrotropic pretreatment. Alkaline peroxide mechanical pulp can be fractionated into cellulose-rich solid fraction to produce sugar and LCNF. The metal ions exhibited excellent catalytic activity and obviously enhanced the degree of delignification, especially when using CuCl_2_. The confocal images highlighted the lignin redistribution on the fiber walls, which affected the downstream production. The pretreated APMP fiber with the lower lignin content was enzymatically hydrolyzed to produce sugars with higher enzymatic efficiency and mechanically fibrillated to produce LCNF with lower height. This work demonstrated that the metal chloride-assisted hydrotropic process has potential applications for low-cost fractionation of lignocellulosic biomass.

## Figures and Tables

**Table 1:** Chemical composition of raw material and metal chloride-assisted hydrotropic pretreated materials along with byproduct concentration in pretreatment spent liquor.

Sample Abbreviation ^1^	Solid					Spent Liquor	
	Glucan (%)	Xylan (%)	Acid Soluble Lignin (%)	Klason Lignin (%)	Solid Yield (%)	Glucose (g/L)	Xylose (g/L)
**APMP**	46.07	16.03	2.92	17.73	100	–	–
**P**	51.18	14.70	1.07	12.13	80.18	ND	1.1
**P–FeCl_3_**	53.31	13.02	0.67	10.80	65.94	0.2	1.6
**P–AlCl_3_**	58.59	13.22	0.83	9.17	63.32	0.1	1.6
**P–CuCl_2_**	61.64	13.02	0.74	9.03	64.47	0.2	1.7

^1^ APMP and P stand for alkaline peroxide mechanical pulp and *p*-TsOH hydrolysis, respectively.

**Table 2:** List of morphological, crystallinity index, surface charge, and thermal properties of the resulting lignocellulosic nanofibrils.

Sample Abbreviation	Average Height (nm)	CrI (%)	Zeta Potential (mV)	T_max__Ⅰ_ (°C)	T_max__Ⅱ_ (°C)
**APMP**	–	53.8	–		368.7
**P–LCNF**	25.2	44.3	–31.4 ± 1.7	197.0	282.0
**P–FeCl_3_–LCNF**	15.1	44.7	–30.7 ± 1.7	197.2	282.2
**P–AlCl_3_–LCNF**	11.5	47.8	–33.7 ± 2.7	195.5	280.5
**P–CuCl_2_–LCNF**	9.0	51.9	–35.4 ± 1.8	197.6	282.6
